# Methods of Modification of Mesenchymal Stem Cells and Conditions of Their Culturing for Hyaline Cartilage Tissue Engineering

**DOI:** 10.3390/biomedicines9111666

**Published:** 2021-11-11

**Authors:** Maria V. Shestovskaya, Svetlana A. Bozhkova, Julia V. Sopova, Mikhail G. Khotin, Mikhail S. Bozhokin

**Affiliations:** 1 Institute of Cytology of the Russian Academy of Sciences, Tikhoretsky Ave. 4, 194064 St. Petersburg, Russia; marieshestovskaya@gmail.com (M.V.S.); y.sopova@spbu.ru (J.V.S.); h_mg@mail.ru (M.G.K.); 2 Vreden National Medical Research Center of Traumatology and Orthopedics, Academica Baykova Str., 8, 195427 St. Petersburg, Russia; clinpharm-rniito@yandex.ru; 3 Center of Transgenesis and Genome Editing, St. Petersburg State University, Universitetskaja Emb., 7/9, 199034 St. Petersburg, Russia

**Keywords:** cartilage, tissue engineering, regeneration, repair, scaffolds, stem cells, growth factors

## Abstract

The use of mesenchymal stromal cells (MSCs) for tissue engineering of hyaline cartilage is a topical area of regenerative medicine that has already entered clinical practice. The key stage of this procedure is to create conditions for chondrogenic differentiation of MSCs, increase the synthesis of hyaline cartilage extracellular matrix proteins by these cells and activate their proliferation. The first such works consisted in the indirect modification of cells, namely, in changing the conditions in which they are located, including microfracturing of the subchondral bone and the use of 3D biodegradable scaffolds. The most effective methods for modifying the cell culture of MSCs are protein and physical, which have already been partially introduced into clinical practice. Genetic methods for modifying MSCs, despite their effectiveness, have significant limitations. Techniques have not yet been developed that allow studying the effectiveness of their application even in limited groups of patients. The use of MSC modification methods allows precise regulation of cell culture proliferation, and in combination with the use of a 3D biodegradable scaffold, it allows obtaining a hyaline-like regenerate in the damaged area. This review is devoted to the consideration and comparison of various methods used to modify the cell culture of MSCs for their use in regenerative medicine of cartilage tissue.

## 1. Introduction

Millions of people all over the world face the problems of musculoskeletal diseases and injuries. Damage to the hyaline cartilage of large joints, specifically the knee and the hip, occupies a special place among these problems. There are various causes for articular cartilage such as injuries and excessive physical stress (sports) [1,2], genetic predispositions [3], systemic inflammatory diseases, such as rheumatoid arthritis (RA) [4] and degenerative joint diseases, such as osteoarthritis (OA) and osteochondritis dissecans (OCD) [4,5]. Degenerative lesions of the articular cartilage are observed in 50% of the adult population over 50 years of age [6]. They have a significant negative impact on the quality of life and lead to disability for 10–20% of people over the age of 60 [7,8]. The pronounced deformity caused by osteoarthritis may require arthroplasty. Though this procedure is highly invasive, risky, and very costly [9], the rate of its implementation is growing steadily throughout the world [10,11,12]. Currently, hyaline articular cartilage is considered to have a limited ability for regeneration due to a lack of blood supply combined with significant mechanical stress [13].

Currently, there are many technologies available to surgeons for replacing the damaged layer of the hyaline cartilage [14], the problem of restoring the surface of the articular cartilage has not yet been fully solved [15,16]. One promising approach to replacing defects in the surface layer of large joints is provided by cell and tissue engineering technologies [17,18]. In our opinion, the key factor for obtaining functional regeneration consists in specialized modification of the applied cell culture implanted into the site of a hyaline cartilage lesion [18].

Presently, no standard for preparing an effectively modified cell culture (as well as requirements for its standardization) for its subsequent use in replacing hyaline cartilage defects has been developed. This work is devoted to a review of the currently used methods for modifying cell cultures and conditions for culturing cells for hyaline cartilage repair and a comparative analysis of their advantages and disadvantages.

## 2. Tissue Engineering Approaches to Cartilage Repair

At the end of the 20th century, Dr. R. Langer and Dr. J.P. Vacanti [19] pioneered the technology of creating vascularized tissue scaffolds on the basis of biocompatible and biodegradable synthetic polymers with the potential to grow *de novo* tissues and organs and founded a fundamentally new field that combines the principles of engineering and life sciences, specifically tissue engineering (TE).

At present TE makes use of cell cultures, biodegradable scaffolds, methods of bioengineering, and physical and biological factors for creating functional biological constructs to replace (regenerate), support, and repair damaged tissues [20]. In our opinion, the modification of cell cultures for defect replacement is one of the key stages of the TE approach to tissue and organ regeneration. Tissue engineering provides a promising alternative to traditional methods of articular cartilage regeneration because it allows one to create a new autologous tissue in vitro, similar in its biomechanical characteristics to native hyaline cartilage [7].

The key points in the creation of autologous cartilage transplant are the choice of the cell culture and effective method of its modification aimed to enhance the synthesis of target proteins of the extracellular matrix of hyaline cartilage, and to improve the biomechanical characteristics of the regenerate. Early TE works involved the use of “natural” mechanisms of cell modification, for example, replacement of cultured chondrocytes by progenitor cells, viz. mesenchymal multipotent stromal cells from which chondrocytes differentiate. Researchers assumed that stem cells, once in the damage site, would spontaneously (without additional external stimuli) form a hyaline-like regenerate of a suitable structure [21].

### 2.1. Mesenchymal Stem Cells

The concept of stem cells emerged at the end of the 19th century as a theoretical postulate explaining the ability of certain tissues to undergo self-renewal throughout the entire life of an organism [22]. In 1867, pathologist J. Cohnheim [23] noticed that non-hematopoietic stem cells are present in the bone marrow and may serve a source of fibroblasts involved in wound healing. M. Tavassoli and W.H. Crosby (1968), who were among the first researchers to study stem cells, discovered the proliferative and osteoblastic potentials of bone marrow cells and their autologous transport to extramedullary sites [22]. Later A. Friedenstein (1976) introduced the term mesenchymal stem cells (MSCs), stating that the bone marrow in the postpartum period is a reservoir of stem cells for mesenchymal tissues [21].

Mesenchymal stem cells feature a high proliferative potential and the ability for self-renewal and tissue regeneration, which are important properties for modern tissue engineering and gene therapy [24]. Due to the mesenchymal origin of chondrocytes, they were decided to be replaced by MSCs [20].

At the end of the 20th century, researchers believed that the use of MSCs could solve the problem of de-differentiation (where cells develop from more differentiated to less differentiated state), and also ensure enhanced production of extracellular matrix proteins compared to chondrocytes and thus solve the therapeutic task of restoring the hyaline layer of the articular cartilage [21].

In fact, the use of MSCs for hyaline cartilage regeneration originates from the introduction into clinical practice of microfracture procedure [25], which requires the natural migration of bone marrow mesenchymal stem cells (BM-MSCs) into the defect area after multiple microperforation of the subchondral bone [26]. Along with bone marrow, other sources of MSCs were found, including muscles, dermis, cancellous bone, adipose tissue, periosteum, pericytes, blood, synovium, and other tissue types [24,27]. A number of European studies and clinical trials showed that bone marrow is used as the most common cell source of MSCs due to the renewability of its cellular resource; however, it may not be the most optimal, considering the cell preparation procedure in combination with the differentiation potential and the persistence of the therapeutic effect [28,29,30,31,32]. The second most popular cell source is the adipose derived stromal vascular fraction (ADSVF) because it is abundant in the body and easily available [33].

As far back as the 1980s, evidence was published showing that cells isolated from the postnatal bone marrow of mammals are able, when implanted in vivo, to differentiate into specific cells of the mesenchymal (bone cartilage) tissue [34]. In 1994 S. Wakitani et al. [35] proposed that MSCs to be used to repair large articular cartilage defects. Since MSCs are bone cartilage progenitor cells, the use of various transcription factors and cytokines, for example transforming growth factor β3 (TGF-β3), can direct (enhance) their differentiation in the chondrogenic direction (see Section 2.3.2 for more information) [36,37]. Mesenchymal stem cells can act both directly (due to their ability to differentiate) and indirectly, producing and secreting many factors that enhance the potential of endogenous regeneration of damaged tissue [38].

Among the advantages of MSCs is that their isolation, in contrast with chondrocytes, does not lead to additional traumatization of the intact articular cartilage [37]. Furthermore, the low immunogenicity of autologous MSCs makes cell transplantation well-tolerated by the recipient organism, and a single MSC transplant is safe and does not induce an immune response [38]. Allogeneic MSCs have theoretical advantages over autologous ones in terms of simplifying collection, storage, testing, and implantation procedures. In our opinion, the future of hyaline cartilage cell engineering consists in the use of safe and standardized allogeneic cell products, but today there are concerns about the cell survival and viability rates, as well as immune rejection [38], and at present this issue is debatable [39,40].

C.H. Jo et al. [41] provided evidence for the efficacy of intra-articular MSC injections for cartilage regeneration in OA over a medium-term horizon. F. Davatchi et al. [42] reported a medium-term improvement in the condition of the knee joint in OA patients after the intra-articular injection of immunophenotyped BM-MSCs. An improvement in clinical parameters according to the VAS (Visual Analog Scale) and PGA (Patient Global Assessment) was recorded with a follow-up period of up to 5 years. In the long term, the efficacy of allogeneic MSCs cultured in 3D hyaluronic acid (HA) hydrogel was demonstrated for Kellgren–Lawrence Grade III osteoarthritis patients, as well as patients with ICRS (International Cartilage Repair Society) Grade 4 cartilage defects [43]. The authors found a stable improvement in the VAS and IKDC (International Knee Documentation Committee) scores over 7 years of follow-up. Histological analysis of biopsies taken after 1 year showed that the structure of the regenerate corresponded to hyaline cartilage, and MRIs taken after 3 years indicated the integrity of the regenerate. However, this technique required a long rehabilitation period.

Since the pool of multipotent cells is small, and their number, especially in adult patients, is limited (with a tendency to decrease with age), their isolation is associated with certain difficulties. An alternative to MSCs appeared when Thomson (1998) discovered pluripotency, that is the ability of cells to differentiate into all cell types, except for cells of extraembryonic organs [44].

Embryonic stem cells (ESCs) were the first described pluripotent cells. The chondrogenic potential of ESCs was first discovered in teratomas, where cartilage islets are grouped together with other tissue types in a chaotic manner. Embryonic stem cells originate from embryoblasts at the blastocyst stage and feature a high proliferative activity in an undifferentiated state throughout a long period of culturing [45]. However, the biggest disadvantage of using ESCs lies in their key advantage, that is high but, unfortunately, uncontrollable proliferative potential. At present ESCs are not used in clinical practice because of the high risk of teratoma formation [46].

A more recent development is induced pluripotent stem cells (iPSCs), derived from the epiblast layer of implanted embryos, and induced by four growth factors: Oct4, Sox2, Klf4, and c-Myc (Yamanaka cocktail) [47]. These cells, in contrast with ESCs, have an unlimited ability for self-renewal, and their use is safe and associated with no ethical problems [48,49]. Recently, iPSCs showed encouraging results in in vitro cartilage regeneration experiments [50]. However, the cartilage generated by iPSCs was a heterogeneous combination of hypertrophic, articular, and fibrous cartilage [51]. Thus, it remains unclear whether the *de novo* formed regenerate possesses the mechanical and functional properties of the native articular cartilage. In our opinion, this is due to the fact the proliferative potential of this subpopulation of cells is quite difficult to MSCs. Moreover, to create autologous iPSCs and transplant them into a patient, a complicated biotechnological problem must be solved for each patient and this is quite expensive [52].

More research is needed to rule out the risks of carcinogenic and tumorigenic effects [53]. Until now the use of MSCs, despite the existing disadvantages (Table 1), remains the prevailing direction in the tissue engineering of hyaline cartilage due to the absence of strict legislative restrictions, relative ease of isolation and culturing, no need for complex and directed differentiation, and enhanced proliferative potential.

Indeed, the tissue differentiated from MSCs can be classified as a cartilage tissue, since it expresses some proteins characteristic and important for hyaline cartilage, such as type II collagen and aggrecan [29]. However, the component ratio of the extracellular matrix (ECM) is generally not the same that of native hyaline articular cartilage, which, in our opinion, has a negative impact on the mechanical characteristics of the *de novo* regenerate [54,55]. Apparently, this is since the presence of MSCs in the hyaline cartilage is a necessary but insufficient condition for the formation of a full-fledged hyaline-like regenerate, and additional conditions and/or targeted and specialized modification are required to successfully solve this complex problem.

The ability of MSCs to differentiate in a targeted manner and maintain the chondrogenic phenotype is closely related to the local microenvironment, mechanical stress, and need to maintain a certain morphological structure. Obviously, in addition to the local microenvironment, the quality and further proliferation of the resulting MSCs depend on the isolation method and the state of the donor’s (gender, age, genetic characteristics and, concomitant diseases) [56], as well as other unknown factors. It is known that the MSCs from patients suffering from diabetes, obesity, rheumatoid arthritis, and systemic inflammatory diseases partially lose their therapeutic function and the potential for proliferation and differentiation [57].

Thus, the use of MSCs in clinical practice now faces a number of limitations, including attenuation of the therapeutic effect under the influence of endo- and exogenous factors, lack of an optimized protocol for MSC isolation and ex vivo preparation for clinical use, and difficulty in reproducing the spatial organization of the native cartilage and, as a consequence, deterioration of the mechanical properties of regenerate [53,56,57]. Furthermore, as already mentioned, even the most successful results of chondrogenic differentiation of MSCs do not ensure that the biochemical composition allows the ECM to withstand prolonged and significant mechanical stress [54,55].

For correct differentiation and functioning of MSCs, a natural 3D environment should be created, where: (i) correct intercellular interaction is ensured; (ii) signal transmission with ECM components is possible; (iii) the growth factors produced by the cells form a dose-dependent concentration gradient without “forced” polarization/organization; and (iv) ECM fibrils restrict cell proliferation and promote correct spatial distribution of the secreted growth factors [58]. Furthermore, in our opinion, during MSC differentiation, the cultured cells should be subjected to mechanical exposure, which, most likely, is necessary to correctly organize the hyaline cartilage [53].

### 2.2. Three-Dimensional Environment and Scaffolds

In parallel with the choice of cell culture, researchers focused on the conditions for its proliferation [25,59,60,61]. It was noted that 3D cell culturing repeats the natural conditions of cell proliferation in the body and allows one to obtain a more functional regenerate; as a result, three-dimensional biomimetic structures—scaffolds—were created, which can be considered as one of the main tools for modifying cell culturing conditions. The use of scaffolds in hyaline cartilage regeneration offers a number of advantages: the possibility of reliably fixing the cell-engineered construct (CEC) on the surface of the damaged joint area, protection from significant mechanical stress at the initial stage after implantation, and, most importantly, a natural 3D microenvironment. The 3D structure induces spontaneous cell proliferation in the chondrogenic direction to generate an extracellular matrix characteristic of the hyaline cartilage and drives the synthesis of key proteins, such as type II collagen and aggrecan, thereby preventing cell de-differentiation [40,53,58,61,62].

However, at present there are also scaffold-free technologies, which involve the implantation into the defect area not of individual cellular elements, but of their aggregates, specifically cell spheroids [63]. To form spheroids, MSCs are cultured under conditions that prevent their adhesion to a solid surface. At present the following technologies are used [64]: pellet culture (concentration by precipitation and dispersion of the cell aggregate over U-shaped wells) [65]; liquid overlay (forming a film of a non-adhesive material, such as agarose, on plastic to prevent cell attachment) [64,66]; (hanging drop (culturing in a plate and its further turning upside down) [67]; spinner flask culture (continuous stirring) [68]; rotating way vessel [69]; microfluidics or “spheroids-on-chip” (microwells, microstructures, drop generators, etc.) [64,70]; and magnetic levitation (addition of magnetic particles to the culture to make cells to levitate) [64,71].

The formation of spheroids occurs due to the synthesis and accumulation of the extracellular matrix in the MSC culture: the encapsulation of MSCs takes place in the self-synthesized ECM [72]. The formed spheroids are implanted in the defect site and then special physiotherapeutic procedures are performed to promote cell integration into the cartilage tissue [73,74]. In our opinion, this procedure is technically simple and has understandable and predictable consequences, which attracts practicing specialists.

Scaffolds can be fabricated from synthetic biomaterials or a natural extracellular matrix (Table 2).

Biomaterials for tissue engineering should be biodegradable, have a controlled chemical composition and have a porosity to allow optimal cell adhesion, migration, and production of endogenous ECM components [20,95,96,97]. Another important characteristic of the scaffold is stiffness, that is, the ability to resist mechanical deformation [98,99]. Engler et al. were the first to explore the effect of the scaffold stiffness parameter on MSCs and showed that stiff substrates promote the development of a highly organized cytoskeleton with a fusiform morphology, while soft substrates promote the development of a rounded chondrocyte-like morphology and enhanced expression of chondrogenic markers [96].

Later evidence was obtained showing that decreased scaffold stiffness resulted in increased levels of SOX9, type II collagen, aggrecan, as well as oligomeric matrix cartilage protein (COMP) in MSCs [100,101,102]. Thus, in choosing the scaffold, one should bear in mind that the softer the hydrogel substrate, the more likely its deformation during implantation, and the harder it is, the less it meets the conditions for correct chondrogenic differentiation [76,100,101,102].

According to [62,75,76,103,104,105], promising results in articular cartilage regeneration studies were obtained only by those authors who used hybrid (mixed) scaffolds, which we consider quite logical, because hyaline cartilage has a well-defined zoning resulting from the different and strictly defined functions of each layer.

Natural polymers are widespread and biodegradable but inferior in mechanical properties to synthetic polymers. Alginate and chitosan are poor in terms of stiffness/elastic modulus but crosslinking with silk fiber or synthetic polymers (PLA [106], PGA [107], PEG [108]) compensates for this disadvantage [95]. Moreover, alginate lacks adhesion ligands, and its functionalization is achieved with molecules that mimic ECM, the most commonly used of which are RGD [83] and gelatin [92]. Any kind of polymer can also be improved to enhance chondrogenesis by including native ECM molecules (collagen, hyaluronic acid, etc.). Combining synthetic materials to ensure mechanical stiffness and natural components to ensure correct chondrogenesis and to retain a proper phenotype increases the probability of regeneration of the hyaline cartilage in the damaged area. In addition, by varying the structure of the scaffold one can selectively control proliferation, but this is not an easy task [109].

One of the scaffold methods widely used in clinical practice for hyaline cartilage restoration is the implantation of autologous cells (initially chondrocytes, now MSC predominantly) seeded in a scaffold for Autologous Matrix-Induced Chondrogenesis (AMIC) [110]. The essence of the technique is as follows: small areas of the damaged articular surface are pretreated, the defective hyaline cartilage is removed, and then microfracture is performed followed by coating of the defect with a biodegradable membrane/scaffold [111]. As a result, autologous mesenchymal multipotent cells (with an increased proliferative potential) migrate from the subchondral bone through the microfracture holes into the 3D scaffold. A multilayer fixable scaffold (or membrane) allows the cell culture to proliferate under 3D conditions [112,113], and its structure favors the formation of a regenerate resistant to external stress in the mid-term perspective [114,115]. As examples of such scaffolds used in clinical practice, we can mention Chondro-Gide^®^ [116,117], NOVOCART^®^ 3D [118,119,120,121,122], MaioRegen [118,123,124], Agili-C™ [118,125,126], BioSeed^®^-C [118,127,128,129], etc.

The AMIC technique does not require compliance with strict legal and ethical standards for cell manipulation and is simple, economically accessible, and allows autologous 3D culturing of MSC progenitor cells in the defect area without a preliminary in vitro culturing step [130,131].

Currently, a new 3D bioprinting technology which is used to create CEC based on the MSCs culture and a biodegradable scaffold has become available [132]. This procedure is technologically complicated, and it is necessary to select the optimal conditions for bioprinting, including the composition of the polymer, bio-ink and the use of various stimuli for cell modification [133,134]

3D bioprinting makes it possible to distribute cells accurately and evenly within the scaffold, supplement CEC with the necessary factors for chondrogenic proliferation of hyaline cartilage, for example, recombinant proteins, and also indirectly affects cell proliferation in the chondrogenic direction. [133,135]

In our opinion, 3D bioprinting using MSCs for tissue engineering of hyaline cartilage has been dynamically developing in recent years and, in the nearest future, will become the main method for creating CECs used for tissue engineering of hyaline cartilage. However, a detailed review of all issues related to 3D bioprinting of hyaline cartilage using MSCs is clearly beyond the scope of this work.

To produce a hyaline-like regenerate from an MSC culture and a 3D scaffold is a difficult task; the problem of targeted differentiation and formation of a regenerate with native functions is impossible to solve completely using indirect modification methods, even though a great number of different models of microenvironments to control MSC proliferation have been designed at present. In our opinion, much attention should be paid to direct methods of cell modification, which will be discussed in the next section of our review.

### 2.3. Direct Modification of Mesenchymal Stem Cells

The above methods for the modification of cell culturing conditions are indirect only and effective within certain limits. These methods have received wide acceptance due to their simplicity, availability, and relative safety. In our opinion, direct cell modification is a more difficult and yet a more promising and interesting task. By creating an artificial regenerate based on cells with a target-modified proliferative potential, we can program them to generate a transplant with biomechanical properties similar to those of the native hyaline cartilage.

Almost all experimental methods of hyaline cartilage regeneration, including those already introduced in clinical practice on a limited scale, make use, to one degree or another, different direct modification techniques. This difficult procedure is necessary for pointwise alteration of cell proliferation with the aim to enhance the synthesis of the required proteins of the extracellular matrix of hyaline cartilage and thus bring the resulting regenerate in its properties closer to the native (intact) hyaline cartilage.

**Figure 1:**
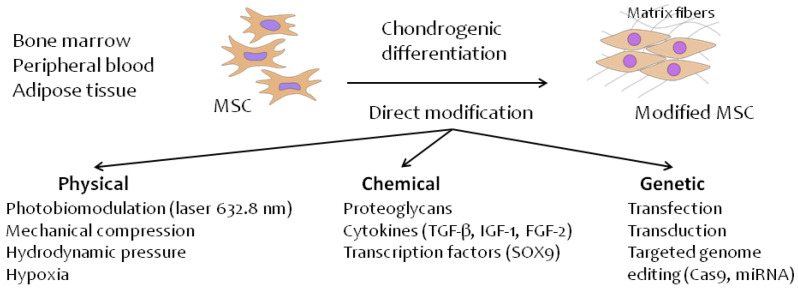
Direct methods of MSC modification for chondrogenic differentiation in hyaline cartilage tissue.

Presently, many physical, chemical and biological factors are known that affect the direction of stem cell differentiation [37,70,136,137,138,139,140,141,142,143,144,145,146]. In principle, these factors can be divided into three groups: physical, chemical, and genetic (Figure 1).

#### 2.3.1. Physical Modification Methods

The effect of physical factors on MSC proliferation is founded on chain of complex and often poorly understood molecular processes that implicate physical phenomena and lead to changes in gene expression, protein synthesis, and intercellular interactions. Research into such phenomena is still in progress and their detailed description is beyond the scope of this review.

Today, the main physical parameters affecting chondrogenic cell proliferation are periodic physical (mechanical) stress, hypoxia, and electromagnetic radiation (photobiomodulation) [136,137,138,139,140,141], and the consequent transformation of the physical signal into a biochemical signal mediated by integrins and the focal adhesion (FA) protein complex [147].

Dynamic mechanical stress affects the proliferation of the articular cartilage and its degeneration processes, altering the production of extracellular matrix proteins and matrix metalloproteinases [139]. Recent studies provided evidence for a positive effect of long-term dynamic compression (after preliminary initiation of chondrogenic differentiation of cells in vitro) on MSC chondrogenesis with enhanced synthesis of type II collagen, aggrecan [148,149], GAG, SOX9 transcription factor [149], and TGF-β1 [150].

**Figure 2:**
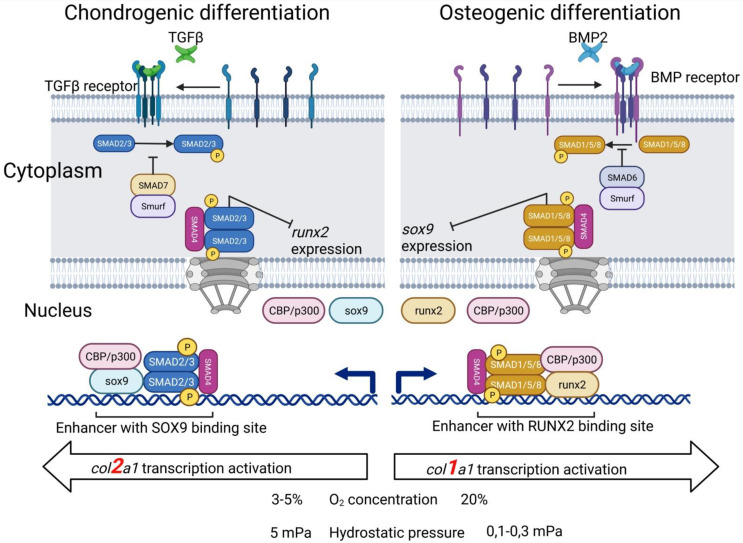
Chondrogenic and osteogenic differentiation of MSCs depends on various conditions and stimuli.

The cartilage ECM is characterized by a high-water content (up to 80%) [151] and low permeability, and consequently, exposure to load creates hydrostatic pressure (HP), which varies depending on the intensity of physical activity from 2 to 18 MPa [141,152]. The physiological levels of HP (5 MPa) significantly enhance the synthesis of the cartilage matrix [152,153], a high hydrostatic pressure (25 MPa) induces proosteoarthritic effects (Col II and aggrecan inhibition, increased expression of metalloproteinases and their synthesis) [154], while a low hydrostatic pressure (100–300 kPa) promotes osteogenic differentiation (increased expression of Runx2, ALP, and osteopontin) [155], resulting in bone tissue from MSCs rather than normal hyaline cartilage being formed (Figure 2).

Hyaline cartilage proliferation occurs under a low (1–3% O_2_) oxygen environment, whereas the normal oxygen level in the air is 23%; therefore, hypoxic conditions can enhance chondrogenesis [156]. Although the hypoxia-induced factor-1α (HIF-1α) is a key mediator of the beneficial effect of a low oxygen environment on chondrogenesis [157], the underlying mechanisms that mediate hypoxic conditions are still unclear. H. Lee et al. [158] and H. Bae et al. [159] reported evidence for increased mRNA expression of type II collagen, aggrecan, and SOX9 transcription factor under hypoxic conditions (1–5% O_2_).

While the correlation between chondrogenic differentiation and a low oxygen concentration and a high physical load is, in our opinion, logically predictable (these are natural conditions of hyaline cartilage proliferation in the body), the effect of electromagnetic radiation on chondrogenic differentiation is not directly obvious. For example, as we showed previously [141], the low-intensity coherent electromagnetic radiation with a wavelength of 638.2 nm significantly increases expression of *tgfb3*, *sox9*, and *col2a1* genes leadings to chondrogenic differentiation of MSCs and initiation aggregation of cellular elements; however, this physical factor had a weaker effect than treatment of the same MSC culture with the recombinant human TGF-β3 protein, a key cytokine responsible for chondrogenesis.

Consequently, the exposure of cell culture and/or CEC to physical factors is important for tissue engineering of MSCs, even though the mechanisms of the changes that occur remain largely unknown, and the biophysical data are recommended to be used when designing CECs to replace hyaline cartilage [160]. An interesting fact is that some physical methods for hyaline cartilage repair had been introduced in clinical practice much earlier than the underlying for molecular mechanisms underlying their effect became understood [141]. In our opinion, the accumulated evidence suggests that the presence of a bioreactor with controlled mechanical load and reduced oxygen concentration is at least desirable, but possibly already imperative to ensure chondrogenic cell proliferation and autologous hyaline cartilage regeneration, which is a challenging technical task.

We consider the choice of physical exposure parameters for directing the proliferation of the cartilage tissue in a desired way to be of no less importance, because the same physical impact with different parameters can act as a “physical switch” between the close but incompatible processes of MSC differentiation in the osteogenic and chondrogenic directions. Nevertheless, some physical methods, for example, low-intensity laser radiation, have already been introduced [161] into clinical practice due to their safety and low invasiveness, lack of legal restrictions, and the ability to locally directly exposure to the hyaline cartilage defective site.

#### 2.3.2. Modification Methods Using Proteins

Further evidence for the fact that MSCs plays an active part in the formation of the dynamic microenvironment of cartilage is provided by the data on their ability to synthesize a wide range of ECM molecules, including fibronectin, collagen(s), glycosaminoglycans, and proteoglycans, as well as various cytokines and growth factors involved in cartilage functioning [162].

In the early 21st century, researchers identified the key regulatory proteins that control cartilage tissue and understood the chain of molecular events that underlie hyaline cartilage proliferation. At the same time, it was proposed to use recombinant proteins for additional modification and to force differentiation of the MSC cell culture in the chondrogenic direction. At present, the modification of MSCs using recombinant growth factors (GF) is one of the simplest, safest, and experimentally and clinically proven approaches to altering cell proliferation [22]. Growth factors are an integral part of the formation of real microenvironment conditions and undoubtedly play a key role in the processes of cell development including chondrogenic differentiation [163].

Currently, a large number of proteins have been described in the scientific literature that in one way or another affect MSC chondrogenesis, but their consideration is definitely beyond the scope of this review. Here we will focus on the most studied, experimentally applied, and fundamentally important families. It is known that the fibroblast growth factors FGF-2, insulin-like growth factor IGF-1, hypoxia factors HIFs, cytokines of the transforming growth factor superfamily TGF-β and associated bone morphogenetic proteins BMP-2,4,6, as well as the SOX9 transcription factor have a direct effect on the chondrogenic differentiation of mesenchymal stem cells [67,141,142,164,165] (Figure 2).

##### SRY-Related HMG-Box Genes (SOX)

Chondrogenesis begins with the differentiation of condensed chondrogenic cells into chondroblasts, the main function of which is the synthesize of the extracellular matrix cartilage [20,166]. The differentiation of MSCs into a chondrogenic clone requires expression of the *sox9* (SRY-box 9) gene, a key chondrogenic transcription factor [166,167,168]. The SOX9 protein cooperatively binds to the SOX5/SOX6 complex on active enhancers and super enhancers associated with hundreds of cartilage-specific genes [169].

Many molecular processes control *sox9* expression in the course of chondrogenesis: BMP [170], TGF-β [171], FGF [172], and hypoxia factors HIFs [173] attenuate or increase it, while Notch [174], retinoic acid [175], and inflammation-related signaling pathways (NFκB-mediated) work exclusively to attenuate expression [176,177]. Identification of the transcription factors that control expression of SOX9, as well as establishment of the exact molecular mechanism of the action of proteins on MSC chondrogenesis, remains a difficult and unresolved problem [178,179].

Im et al. [180] developed a plasmid system for the delivery of *sox*-*5*,-*6*,-*9* into AD-MSCs seeded in polymeric scaffolds and then implanted the resulting complex into the osteochondral defects in rabbit knees. The plasmid stimulated the synthesis of proteoglycans and type II collagen in the cartilage, while *col10a1* expression decreased.

##### Transforming Growth Factor β (TGF-β) Superfamily

The transforming growth factor-β TGF-β superfamily was discovered and characterized by several independent research groups in the 1980s [181]. Ligands of the TGFβ superfamily are divided into two subfamilies In terms of structural and functional features: TGF-βs and BMPs (bone morphogenetic proteins) [182]. TGF-β, activin, myostatin, and growth differentiation factor GDF11 belong to the TGF-β subfamily, while BMP-2, BMP-4, and BMP-7 belong to the BMP subfamily.

Numerous TGF-β subfamily members modulate the selection of MSC clones and the progression of mesenchymal differentiation into specific cells by controlling the expression and activity of key transcription factors [183]. Members of the TGF-β family transmit an external signal by binding to cellular receptors of Ser/Thr protein kinases and activate intracellular SMAD-dependent signaling pathways [184]. TGF-β signaling has an important anti-inflammatory effect on hyaline cartilage [185]: TGF-β1 counteracts proinflammatory interleukin 1 (IL-1) signaling in vivo [186,187] and in vitro [188], and TGF-β3 helps to replenish the number of proteoglycans after their inflammation-induced depletion [189].

Bone morphogenetic proteins (BMPs) play an important role in chondrogenesis [163] by participating in the TGFβ signaling pathways and cytokine–cytokine receptor interaction. In fact, BMP-2 enhances the production of cartilage matrix and blocks IL-1-induced degeneration of cartilage tissue [190], while TGF-β3 itself increases the expression of the col2a1 gene, the main protein of the ECM of hyaline cartilage [191,192]. It was also reported that BMP-7 inhibits cell proliferation but stimulates ECM synthesis in the BM-MSC culture [193].

M.B. Gugjoo et al. provided in vitro experimental evidence showing that the stimulation of MSCs by TGF-β1 (generally in combination with IGF-1) promotes hyaline cartilage repair in an osteochondral defect in rabbits [194,195].

##### Insulin-like Growth Factor-I (IGF-I)

IGF-1 was discovered in 1957 by Salmon and Daughaday [196] who defined it as a “sulfation factor” for its ability to stimulate sulfation of the chondroitin chain in the ECM of rat cartilage. Chondroitin sulfates subsequently bind to core proteins (the protein part of the future proteoglycan) and form aggrecan proteoglycans which are important for cartilage [197].

IGF-1 is considered as one of the most essential growth factors involved in maintaining the integrity of the hyaline cartilage [198,199,200]. Recent in vitro experiments showed that IGF-1 can modulate MSC chondrogenesis, irrespective of whether TGFβ signaling is present or absent [201,202]. IGF-1 can accelerate/inhibit the synthesis of ECM components by enhancing collagen II synthesis, and, on the other hand, it can prevent ECM degradation by decreasing MMP synthesis.

According to the in vitro results of Davies et al., the effect of this cytokine is dose-dependent, that is, different doses of IGF-1 induce different biological responses [203]. High doses of IGF-1 increase cell survival in the cartilage defect site and increase the synthesis of type II collagen, as well as improve integrity of the neocartilage tissue. Low doses of IGF-1 induce significant changes in gene expression in the subchondral bone and promote the formation of tissues with better histological characteristics (the cartilage layer and adjacent subchondral bone better correspond in morphology to native tissues) [203,204]. In combination with TGF-β1, IGF-1 stimulates the restoration of hyaline cartilage in bone cartilage defects in rabbits in vivo [194,195].

##### Fibroblast Growth Factor

Fibroblast growth factor (FGF) was discovered in the bovine brain and pituitary gland in 1973–74s by Armelin and Gospodarowicz [205] and described as a mitogen that initiates DNA synthesis by resting embryonic 3T3 fibroblasts.

There are two key peptides of the FGF superfamily that are involved in hyaline cartilage regeneration: FGF-2 (bFGF/basic FGF) and FGF-18. The former can participate in limited cartilage repair processes and decrease the level of aggrecanase (a matrix metalloproteinase that specifically cleaves aggrecan, one of the main proteins of the ECM of hyaline cartilage [206]). Interestingly, the concentration of FGF-2 in the synovial fluid of osteoarthritis patients is double that of patients with healthy knee joints [207].

L. Xiao et al. [208] studied the effect of blocking the FGF receptor in transgenic mice with overexpressed FGF-2 and found that degenerative changes in the OA cartilage were slowed down due to preventing FGFR binding to FGF-2. T. Yamamoto et al. [209] established that FGF-2 was involved in articular cartilage repair in immature, but not in adult animals. It was also shown that FGF-18 stimulated chondrogenesis and cartilage restoration at individually selected concentrations [210,211,212,213], which like as with IGF-1, points to a dose- dependent effect.

Chuma et al. [214] evaluated the effect of FGF-2 on the proliferation and migration of MSCs in vitro and in vivo. In vitro FGF-2 facilitated the mobilization and migration of replicating BM–MSCs, and a two-week long in vivo treatment led to a restoration of 5 mm articular cartilage defects in rabbits by stimulating MSC recruitment to the damaged site.

##### Proteoglycans

As noted in the previous sections, chondrogenic differentiation of MSCs depends on, among other things, the interaction with ECM components [215]. Proteoglycans are high molecular weight compounds consisting of a core protein and one or more attached glycosaminoglycan (GAG) chains [216]. Proteoglycans play an important role in the regulation of cell-to-cell signaling. For example, heparin sulfate chains interact with several growth factors, including fibroblast growth factors (FGFs) and TGFβ, ensuring their binding to specific receptors on the cell surface. Although growth factors usually interact with proteoglycan GAG chains, members of the TGF-β family also bind to the decorin proteoglycan core protein. The combination of TGF-β with decorin inhibits TGF-β activity [217].

Thus, to trigger and maintain MSC chondrogenesis and drive the synthesis of key proteins of hyaline cartilage ECM, such as type II collagen and aggrecan, it is sufficient to use some key chondrogenic growth factors and add them to the cell culture during the culturing period (or incorporate them in a biodegradable scaffold in advance) both in vitro and in vivo. In our opinion, this modification procedure offers the advantages of simplicity and safety, and evidence for its efficacy has been provided by numerous studies. When using cytokines and growth factors for MSC modification, one should consider the dose-dependent effect of these proteins, because the direction of cell differentiation depends on their concentration.

The main disadvantage of using recombinant proteins is their high cost, which makes these methods much more expensive in the cases of large CEC. It should also be kept in mind that the growth factor(s) should be added to the culture medium at each change, and this significantly increases the consumption of recombinant proteins. Not all cytokines are commercially available, and if the necessity to modify the protein arises (by adding or removing some domain), one has to contract a commercial company or to isolate and purify the protein in-house, that is a separate biotechnical task, and significantly limits the prospects for widespread use of recombinant proteins in clinical practice.

#### 2.3.3. Genetic Modification Methods

An additional way to affect MSCs is genetic modification of cells to stimulate the synthesis of necessary growth or transcription factors [218,219]. Gene modification of cells allows one to control not only chondrogenic differentiation, but also to obtain cells with enhanced production of ECM proteins, which is an important condition to ensure that the resulting regenerate is close in biomechanical properties to native hyaline cartilage.

This group of methods includes three basic approaches: non-viral gene transfer (transfection), viral vectors (transduction) and direct genome editing. All three approaches have been used for cartilage repair in animals, but have only been introduced in clinical practice on a limited scale; at the same time, a number of publications on this subject are available [218,219,220,221,222,223].

The choice between ex vivo and in vivo methods of genetic modification depends on several factors, including the gene to be delivered and the vector. Vectors based on adenoviruses, retroviruses, herpes simplex virus, adeno-associated viruses, and lentiviruses, as well as non-viral vectors are used [223]. In vivo procedures are simpler, cheaper, and less invasive but involve direct injection of viruses into the body, and, therefore, such procedures are subject to strict safety regulation. Ex vivo gene transfer procedures are generally more invasive, expensive, and labor-consuming, but they allow control of transduced cells and safety testing prior to implantation [218,223].

##### Transfection and Transduction

Currently, chondrocytes and mesenchymal stem cells are the types of cells most used for genetic modification [224,225]. Some success with genetic modification has been achieved with all cell types via either transfection [226,227,228,229] or transduction with viral vectors [230,231,232,233,234] based on adenoviruses, retroviruses, and at most promisingly recombinant adeno-associated viruses (rAAVs) [235,236,237].

Mesenchymal stem cells, similar to chondrocytes (and any primary cell culture), are almost immune to transfection with plasmid DNA [238,239,240]. Nevertheless, these cells can be transfected by means of electroporation or lipofection [240]. It is known that commercial lipid reagents, such as FuGENE6 and Lipofectamin [241], increase the efficiency of DNA uptake, and pretreatment of cells with hyaluronidase and a mild detergent has a similar effect [242,243]. In our opinion, the efficacy of the transfection procedure is determined not only by the number of genetically modified cells obtained, but also by whether the following conditions are met simultaneously: high cell survival in culture (low cytotoxicity of the procedure), duration of the effect of genetic modification, and reproducibility of modification results. Only if all stages of genetic modification of cells in vitro have been fulfilled with compliance to all the above conditions, success can be achieved in in vivo experiments and, possibly, in further clinical studies. The problem of low efficacy is especially relevant to direct in vivo gene therapy, and, therefore, ex vivo strategies are usually required, with reimplantation of genetically modified cells into the hyaline cartilage defect site [244].

In contrast, transduction is a natural mechanism for introducing foreign genetic material into cells. It is an effective and proven method for promoting (or inhibiting) the synthesis of required proteins in a selected cell culture or subpopulation. However, the efficacy, duration of action, the possibility of protocol standardization, and low cytotoxicity contrast strongly with the main disadvantage of this technique, specifically legislative restrictions on mass implementation even in limited clinical practice.

At present, the scientific literature contains many examples of MSC transduction to enhance chondrogenesis by increasing the expression of genes responsible for the proliferation of hyaline cartilage (*tgfb3*, *sox9*, *acan*, *igf1*, *bmp4*,*7*, etc.), however, all the reported experiments have been carried out either in vitro or in animals [245] (more in the next chapter, Table 3). Nevertheless, there are clinical trials in progress involving use of genetically modified transduced chondrocytes [230,235,246,247], which, in our opinion, is associated with the choice of terminally differentiated cells as a transduced cell culture, as well as the predicted proliferation of such an altered cell culture.

##### CRISPR-Cas, miRNA, and Other Methods of Targeted MSC Modification

CRISPR/Cas is a new versatile and promising method of genome editing, the discovery of which was awarded the Nobel Prize in Chemistry in 2020. It opens new pathways and opportunities for the effective treatment of OA and other degenerative joint diseases [145,146,147]. The CRISPR/Cas9 prokaryotic system consists of three main components: Cas9 nuclease, crRNA (CRISPR RNA), and tracrRNA (transactivating crRNA). The Cas9 protein in complex with RNA recognizes the complementary sequence of foreign DNA and introduces a double-stranded break into it [248]. This method is used to obtain knockouts for various genes.

In the therapy of inflammatory and degenerative diseases, the IL1-β (inflammation), Has2 (chondrocyte accumulation of aggrecan), miR-140 (chondrocyte homeostasis), Mmp13 (tissue degradation), Foxd1 (transcription factor), and some other genes are the most common targets for knockout [214]. Along with knockout, the CRISPR/Cas technology allows activation of specific gene expression; this approach was successfully applied to produce the ECM proteins in hADSC cells [249] and activate lncRNA GRASLND expression, which in turn activated the synthesis of type II collagen and aggrecan in hMSC cells [250].

Despite the rapid growth in the number of studies over a short period of time and the potential for application in medicine, CRISPR/Cas9, similar to other genome editing tools, has a number of limitations. In addition to ethical problems, there is a possibility of introducing additional mutations into the genome, since Cas9 is not very specific and can introduce double-stranded breaks in non-target DNA sequences [251]. This problem is solved by using a catalytically inactive form of the Cas9 protein fused with a transcription activator to enhance transcription.

Along with gene knockout, another approach to approach to OA gene therapy can be provided using short noncoding RNAs (miRNAs), which affect ECM gene expression. Gurusinghe and Strappe [224] showed that the overexpression of miR-140, miR-21, and miR-675 can stimulate chondrogenesis in cultured MSC cells; however, these studies are still far from complete.

Examples of MSC modification (direct or indirect) for cell engineering of hyaline cartilage have been known since the end of the 20th century. Each subsequent generation of such modification approached features a higher efficacy compared to the previous ones; however, such an improvement was associated with increasing complexity of the therapy. Over the past 10 years, it is the genetic modification of cell culture for use within a cell-engineered scaffolds for the replacement of hyaline defects that has become widely used in in vitro and in vivo experiments due to its effectiveness; however, such cell modification has never been applied in the tissue engineering of hyaline cartilage in humans.

In our opinion, the historical choice of mesenchymal stem cells was inextricably linked with the possibility of their potential clinical use, characteristically increased proliferative potential and ability for natural differentiation into chondrocytes, as well as the ease of their production, isolation, and culturing (or migration in the case of microfracturing). Viral transduction of just MSCs imposes restrictions for clinical use, requires experience in virological experiments with cell cultures, as well as additional studies of the proliferation of genetically modified MSCs, which significantly complicates and increases the cost of the technique. Transfection, while allowing limited clinical application, leads to low efficacy and high cytotoxicity of MSCs [243]. Discussion of all aspects of genetic modification of MSCs (and other cells) for hyaline cartilage repair is undoubtedly beyond the scope of this review; detailed information on this topic can be found in [252,253].

**Table 3:** Timeline of MSC modification methods and results of recent studies for each stage (last 5 years publications).

Method of MSC Modification		Experimental Stage		
		In Vitro Studies	Preclinical Studies (Animals)	Clinical Studies
Microfracture		n/a	+ [254,255,256]	+ MT [257,258,259]
Spheroid-based		+ [260,261,262]	+ [73,262,263,264,265]	-
Scaffold-based		+ [89,265,266]	+ [267,268,269,270]	+ ST [271,272] MT [43]
Direct modification	Physical	+ [141,273,274,275]	+ [276,277,278,279]	+ ST [161,280]
	Chemical (protein)	+ [281,282,283,284]	+ [168,285,286]	+ ST [287]
	Genetic	+ [288]	+ [289]	-

n/a—not applicable, ST—short-term, MT—mid-term clinical results.

Modification of MSCs for tissue engineering of hyaline cartilage, however, has some limitations and advantages, which are displayed below.

Advantages:

(1) allows creating more functional hyaline-like regenerate,
(2) allows creating a cell culture with improved properties (increased gene expression and increased synthesis of extracellular matrix proteins), which affects the future properties of the regenerate,
(3) the use of microfracture and scaffolds makes it possible to modify the conditions for the proliferation of MSCs, to restore the damaged area of the hyaline cartilage, and at the same time, to remove the need for a complex stage of cell cultivation in vitro,
(4) some types of MSC modification make it easy to translate it in clinical practice;

Limitations:

(1) in most cases, this is a complicated biological (biophysical) task that prevents the widespread translation of the procedure into clinical practice,
(2) MSC modification can cause the formation that does not match in its biophysical functions to native hyaline cartilage,
(3) the processes occurring during the modification of MSCs have not yet been fully studied,
(4) some types of MSC modification cannot be translated into clinical practice now due to significant law barriers.

## 3. Conclusions

Despite the fact that tissue engineering of large joints is one of the fastest growing branches of science, to date, most of the methods based on cell engineering technologies are experimental, some of them are under clinical testing, and only a limited list of methods is registered for clinical practice. All the methods of modification of MSCs and the conditions of their culturing analyzed in the review have certain drawbacks and limitations. The analysis of scientific publications shows that the clinical use of various commercial 3D-scaffolds as tools for modifying culturing conditions has demonstrated high medium-term efficacy, which, apparently, will add popularity to this method in real clinical practice. Furthermore, protein and physical methods of MSC modification, which have already been introduced in part into clinical practice, appear to hold the greatest promise. Genetic methods for modifying MSCs, despite their efficacy, have significant limitations and have not yet been developed enough to allow their effectiveness to be tested even in limited groups of patients.

## Figures and Tables

**Table 1:** Stem cells potency in hyaline cartilage tissue engineering.

Stromal Cell Type		Pros	Cons
Mesenchymal stem cells (MSC)		Low immunogenicity (autologous MSC); A large number of sources; Simplicity of collection; Differentiation into chondrocytes (specialized cells of hyaline cartilage)	Immune rejection risks (allogeneic MSC); Relatival injury of collecting MSC cells from a patient
Pluripotent stem cells	Embryotic stem cells (ESC)	Unlimited proliferative potential	Carcinogenicity; Ethical issues; Immune rejection risks; Complexity of directed modification
	Induced pluripotent stem cells (iPSC)	Artificial generation from somatic cells	Carcinogenicity; High cost; Complexity of directed modification

**Table 2:** Types of scaffold materials used in cartilage tissue engineering and their effect on MSC organization and proliferation.

Scaffold Base			Cell Programming (Organization and Proliferation)
Natural (polysaccharides)	Glycosaminoglycans (GAGs)	Sulfated (heparin, chondroitin sulfate)	Facilitating cell migration (even distribution) [75]; Binds to FGF, TGF-βand fibronectin via heparin binding domains (HBDs) [76] and coordinates multiple signaling pathways during chondrogenesis [77,78]
		Non-sulfated (hyaluronic acid/hyaluronan)	Facilitating cell migration [75,79]; Increases the expression of type II collagen, aggrecan and SOX9 [80,81]; Promotes the expression of CD44 [81]
	Alginates		Facilitating cell migration and proliferation (only when biofunctionalization with adhesion ligands: cross-linking with RGD/gelatin/fucoidan [82,83]
	Chitosan		Stimulates GF production due to structural similarity to GAG [84]; Promotes the formation of MSC spheroids [85]
	Cellulose		Partially sulfated cellulose increases the levels of TGF-β3, collagen II [86]; Promotes the synthesis of sulfated GAG and collagen II under biomechanical stimulation [87]
Native (proteins)	Collagen		Facilitating adhesion and phenotype retention [77]; Promotes the biosynthesis of COL2A1, COL1A1, SOX9, collagen II, aggrecan and matrilin-1 [88,89]
	Fibrin (silk/spider silk)		Facilitating adhesion and migration [90]; Promotes the synthesis of glycosaminoglycans and collagen II [91]
	Gelatin		Providing tight intercellular interactions [92]; Promotes the synthesis of glycosaminoglycans (chondroitin sulfates) and collagen II [92,93]
Synthetic	Polylactide (PLA), Polyglutamic acid (PGA)		Proper spatial imitation of cartilage tissue due to good biomechanical properties [89]
	Poly(ethylene glycol) (PEG)		Promoting proliferation and transmission of biochemical signals (only when biofunctionalization with adhesion ligands [83]) [94]

## Data Availability

Data sharing not applicable.

## Abbreviations

RA	Rheumatoid arthritis
OA	Osteoarthritis
OCD	Osteochondritis dissecans
TE	Tissue engineering
MSCs	Mesenchymal stem cells
BMSCs	Bone Marrow Mesenchymal Stem Cells
ADSVF	adipose derived stromal vascular fraction,
TGF-β3	Transforming growth factor β
VAS	Visual analog scale
PGA	Patient Global Assessment
HA	Hyaluronic acid
ICRS	International Cartilage Repair Society
IKDC	International Knee Documentation Committee
MRI	Magnetic Resonance Imaging
ESCs	Embryonic stem cells
iPSCs	Induced pluripotent stem cells
ECM	Extracellular matrix
GAGs	Glycosaminoglycans
HBD	Heparin-binding domens
PLA	Polylactide
PGA	Polyglutamic acid
PEG	Poly(ethylene glycol)
CEC	Cell-engineered construct
COMP	Cartilage oligomeric matrix protein
RGD	Arginine-glycine-asparagine protein sequence
AMIC	Autologous matrix-induced chondrogenesis
FA	Focal adhesion
HD	Hydrostatic pressure
HIF-1α	Hypoxia-inducible factor-1α
GF	Growth factor
SOX	SRY-related HMG-box genes
FGF-2	Fibroblast growth factor
IGF-I	Insulin-like growth factor-I
BMP	Bone morphogenetic proteins
GDF	Growth/differentiation factors
IL-1	Interleukin 1
LAP	Latency-associated peptide
MMP3	Metalloproteinase 3
ROS	Reactive oxygen species
PAM	Protospacer adjacent motif
